# Supplementary material for: Natural Silencing of Quorum-Sensing Activity Protects Vibrio parahaemolyticus from Lysis by an Autoinducer-Detecting Phage
Source: bioRxiv. 2023 Jun 5:2023.06.05.543668. Preprint. [Version 1] doi: 10.1101/2023.06.05.543668 (PMC10274711; doi:10.1101/2023.06.05.543668)
Supplement: Supplement 1 [file NIHPP2023.06.05.543668v1-supplement-1.pdf]

bioRxiv preprint doi: https://doi.org/10.1101/2023.06.05.543668; this version posted June 5, 2023. The copyright holder for this preprint (which was not certified by peer review) is the author/funder, who has granted bioRxiv a license to display the preprint in perpetuity. It is made available under aCC-BY 4.0 International license.

Supplementary Figure 1

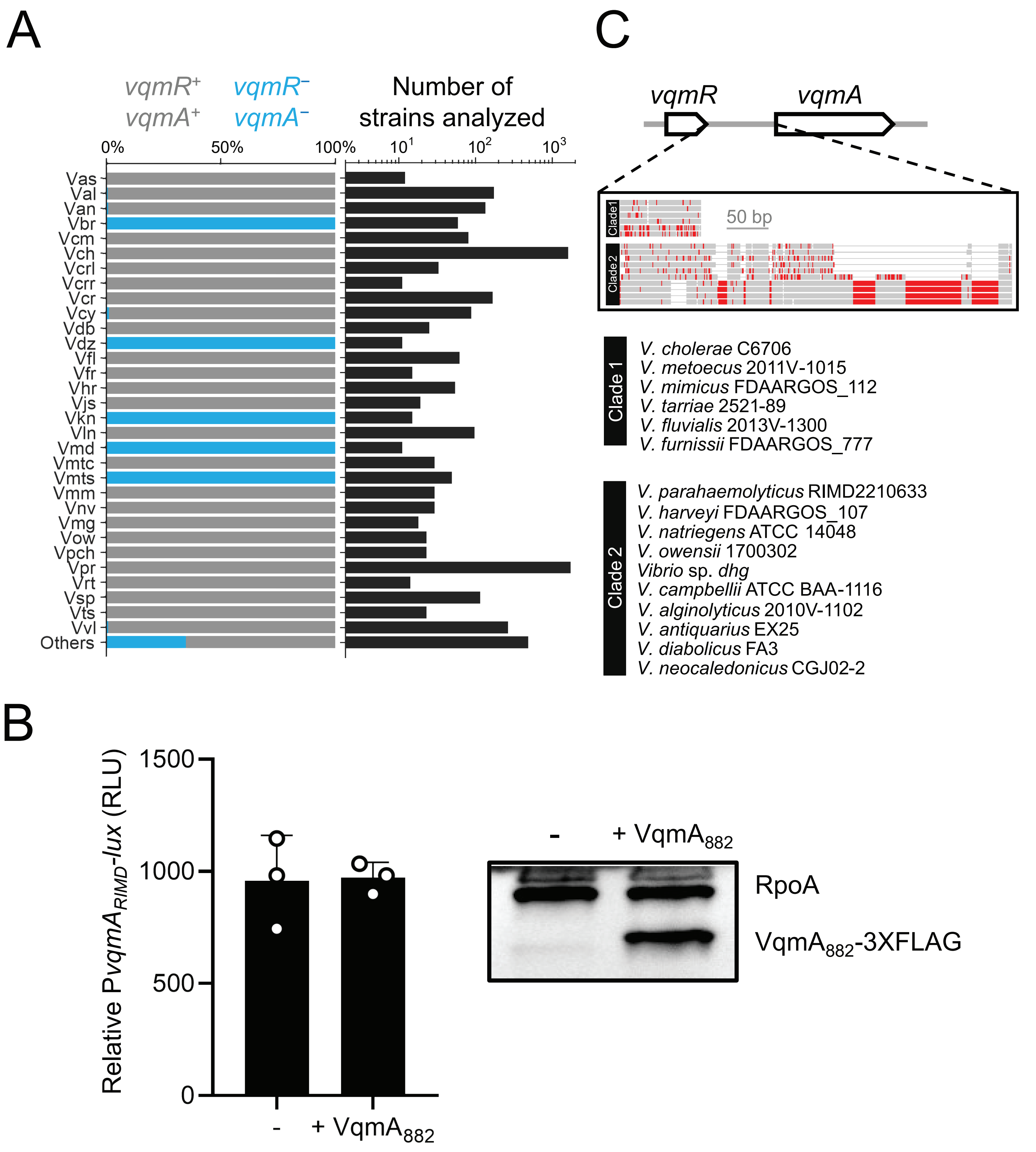

bioRxiv preprint doi: https://doi.org/10.1101/2023.06.05.543668; this version posted June 5, 2023. The copyright holder for this preprint (which was not certified by peer review) is the author/funder, who has granted bioRxiv a license to display the preprint in perpetuity. It is made available under aCC-BY 4.0 International license.

Supplementary Figure 1. *V. parahaemolyticus* strain 882 is the only sequenced *Vibrio* that lacks *vqmR* and harbors *vqmA*, *vqmA* expression is not auto-regulated in *V. parahaemolyticus*, and *Vibrio vqmA* promoters cluster into two distinct classes.

(A) Percentage of strains possessing (gray) or lacking (turquoise) *vqmR-vqmA* pairs (*left*), and number of strains analyzed (*right*) for the designated species. Vas: *Vibrio aestuarianus*. Val: *Vibrio alginolyticus*. Van: *Vibrio anguillarum*. Vbr: *Vibrio breoganii*. Vcm: *Vibrio campbellii*. Vch: *Vibrio cholerae*. Vcrl: *Vibrio coralliilyticus*. Vcrr: *Vibrio coralliirubri*. Vcr: *Vibrio crassostreae*. Vcy: *Vibrio cyclitrophicus*. Vdb: *Vibrio diabolicus*. Vdz: *Vibrio diazotrophicus*. Vfl: *Vibrio fluvialis*. Vfr: *Vibrio furnissii*. Vhr: *Vibrio harveyi*. Vjs: *Vibrio jasicida*. Vkn: *Vibrio kanaloae*. Vln: *Vibrio lentus*. Vmd: *Vibrio mediterranei*. Vmtc: *Vibrio metoecus*. Vmts: *Vibrio metschnikovii*. Vmm: *Vibrio mimicus*. Vnv: *Vibrio navarrensis*. Vmg: *Vibrio nigripulchritudo*. Vow: *Vibrio owensii*. Vpch: *Vibrio paracholerae*. Vpr: *Vibrio parahaemolyticus*. Vrt: *Vibrio rotiferianus*. Vsp: *Vibrio splendidus*. Vts: *Vibrio tasmaniensis*. Vvl: *Vibrio vulnificus*.
(B) *Left*: Relative P*vqmA*$_{RIMD}$*-lux* output from *E. coli* carrying arabinose-inducible *vqmA*$_{882}$*-3XFLAG*. The treatments - and + VqmA$_{882}$ refer to water and 0.2% arabinose, respectively. RLU as in Figure 2B. *Right*: representative western blot of VqmA$_{882}$-3XFLAG produced by the *E. coli* in the left panel. RpoA was used as the loading control.
(C) Multiple DNA sequence alignment of the intergenic regions between *vqmR* and *vqmA* for the *V. cholerae* clade (Clade 1) and the *V. parahaemolyticus* clade (Clade 2). A representative strain (as designated) was chosen for each species in each clade. Thick gray or red bars indicate, respectively, nucleotides that are identical with or different from the consensus (>50% agreement among aligned sequences). Thin gray lines indicate gaps in the sequence alignments. Scale bar indicates 50 bp.
Data in B are represented as means ± std with *n* = 3 biological replicates (*left*) and representative of two independent experiments (*right*).

bioRxiv preprint doi: https://doi.org/10.1101/2023.06.05.543668; this version posted June 5, 2023. The copyright holder for this preprint (which was not certified by peer review) is the author/funder, who has granted bioRxiv a license to display the preprint in perpetuity. It is made available under aCC-BY 4.0 International license.

Supplementary Figure 2

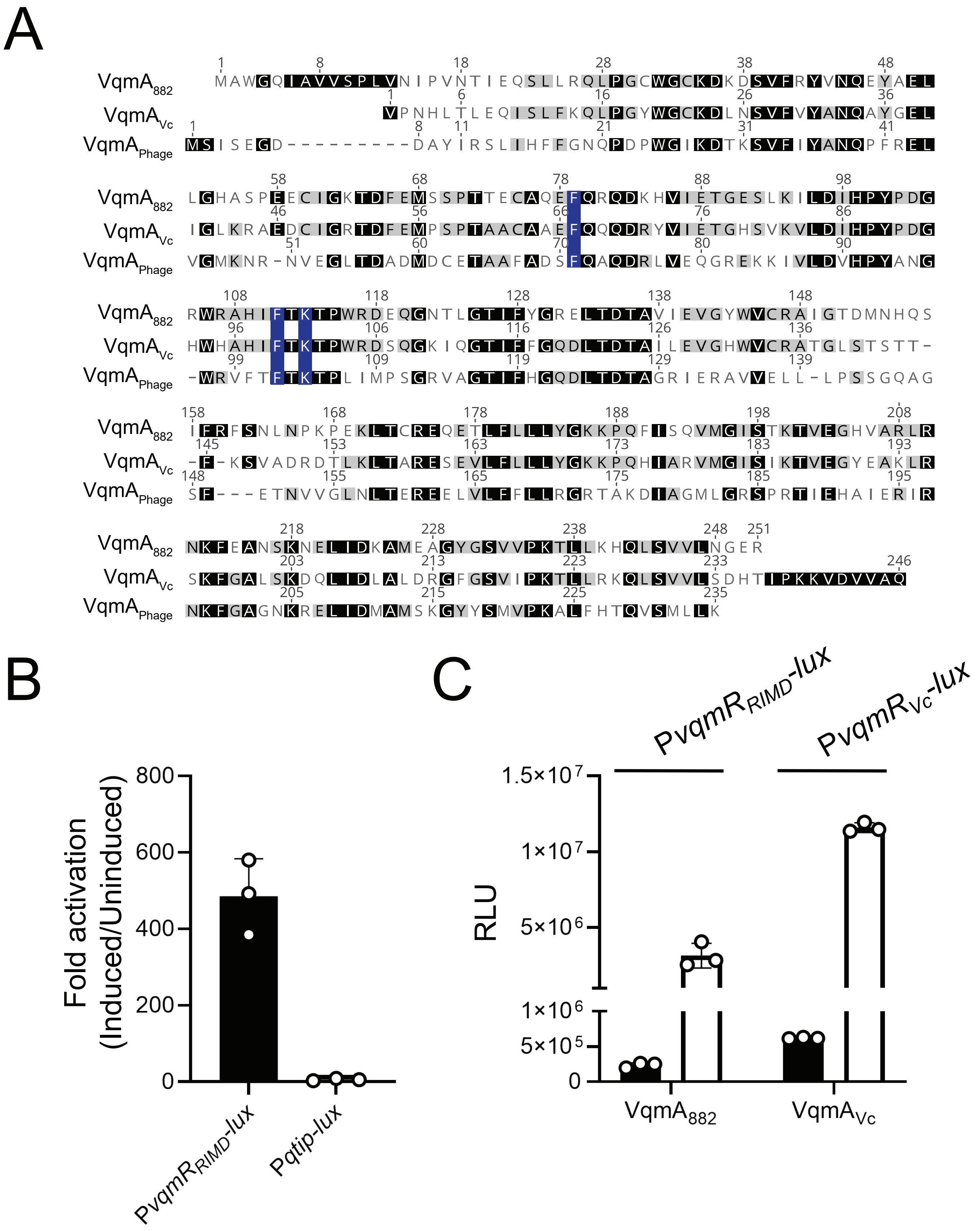

bioRxiv preprint doi: https://doi.org/10.1101/2023.06.05.543668; this version posted June 5, 2023. The copyright holder for this preprint (which was not certified by peer review) is the author/funder, who has granted bioRxiv a license to display the preprint in perpetuity. It is made available under aCC-BY 4.0 International license.

Supplementary Figure 2. $VqmA_{882}$ binds DPO and promoter DNA.

(A) Protein sequence alignment (ClustalW) showing *V. parahaemolyticus* strain 882 VqmA ($VqmA_{882}$), *V. cholerae* VqmA ($VqmA_{Vc}$), and phage VP882 VqmA ($VqmA_{Phage}$) proteins. Black and gray boxes designate identical and conserved residues, respectively. Numbering indicates amino acid positions. Blue boxes indicate key conserved DPO-binding residues from $VqmA_{Vc}$ (F67, F99, and K101).
(B) Relative fold activation of P$vqmR_{RIMD}$-*lux* or P*qtip-lux* from Δ*tdh E. coli* harboring arabinose-inducible $vqmA_{882}$-*3XFLAG.* Fold activation was calculated by dividing the RLU of induced cells (0.02% arabinose and 10 µM DPO) by the RLU of uninduced cells.
(C) Relative P$vqmR_{RIMD}$-*lux* and P$vqmR_{Vc}$-*lux* from Δ*tdh E. coli* harboring arabinose-inducible $vqmA_{882}$-*3XFLAG* (designated $VqmA_{882}$) or $vqmA_{Vc}$-*3XFLAG* (designated VqmAVc), respectively. *E. coli* were treated with either water (black bars) or 10 µM DPO (white bars). All cells were treated with 0.02% arabinose.
Data are represented as means ± std with *n* = 3 biological replicates (B, C). RLU as in Figure 2B (B, C).

bioRxiv preprint doi: https://doi.org/10.1101/2023.06.05.543668; this version posted June 5, 2023. The copyright holder for this preprint (which was not certified by peer review) is the author/funder, who has granted bioRxiv a license to display the preprint in perpetuity. It is made available under aCC-BY 4.0 International license.

Supplementary Figure 3

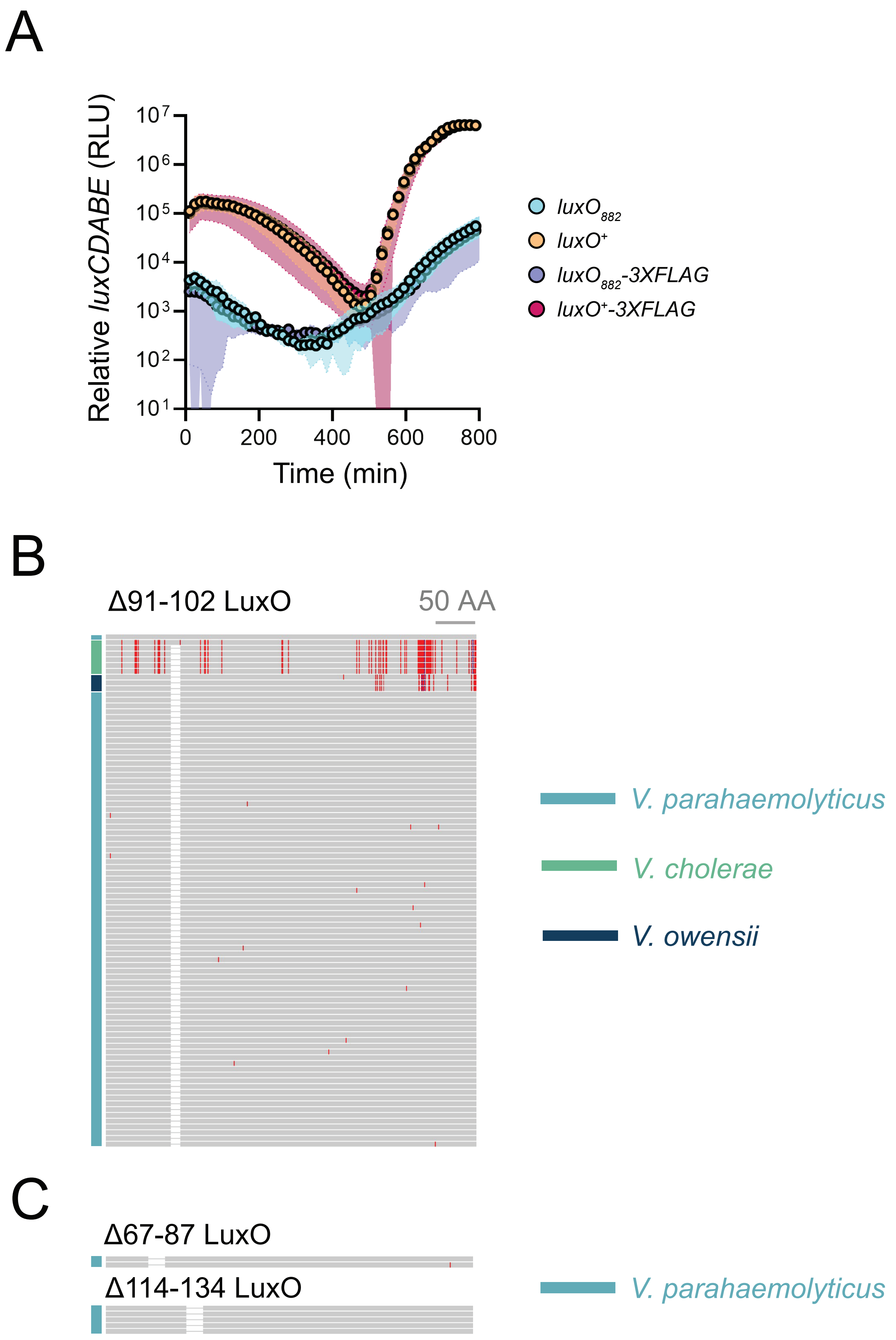

bioRxiv preprint doi: https://doi.org/10.1101/2023.06.05.543668; this version posted June 5, 2023. The copyright holder for this preprint (which was not certified by peer review) is the author/funder, who has granted bioRxiv a license to display the preprint in perpetuity. It is made available under aCC-BY 4.0 International license.

Supplementary Figure 3. LuxO$_{882}$-3XFLAG and LuxO^{+}-3XFLAG are functional and 88 *Vibrio* strains possess the Δ91-102 LuxO deletion.

(A) Relative *luxCDABE* output over time from the 882 *luxO$_{882}$* (cyan), 882 *luxO^{+}* (orange), 882 *luxO$_{882}$-3XFLAG* (purple), and 882 *luxO^{+}-3XFLAG* (pink) strains. Data are represented as means ± std with n = 3 biological replicates.
(B) Multiple amino acid sequence alignment of LuxO in *Vibrio* strains that carry the Δ91-102 *luxO* mutation. Gray or red vertical bars indicate, respectively, amino acids that are identical to or different from the consensus (>50% agreement among aligned sequences). White boxes indicate the 91-102 amino acid deletion. Blue vertical lines indicate insertions. Teal indicates *V. parahaemolyticus* strains, green indicates *V. cholerae* strains, and dark blue indicates *V. owensii* strains. Scale bar indicates 50 amino acids (abbreviated AA). All sequences are aligned with respect to the LuxO sequences of *V. parahaemolyticus* RIMD2210633 and *V. cholerae* C6706, which are shown in the first and second row, respectively.
(C) As in (B), except the strains carry the Δ67-87 (top) or Δ114-134 (bottom) *luxO* mutation.

bioRxiv preprint doi: https://doi.org/10.1101/2023.06.05.543668; this version posted June 5, 2023. The copyright holder for this preprint (which was not certified by peer review) is the author/funder, who has granted bioRxiv a license to display the preprint in perpetuity. It is made available under aCC-BY 4.0 International license.

Supplementary Figure 4

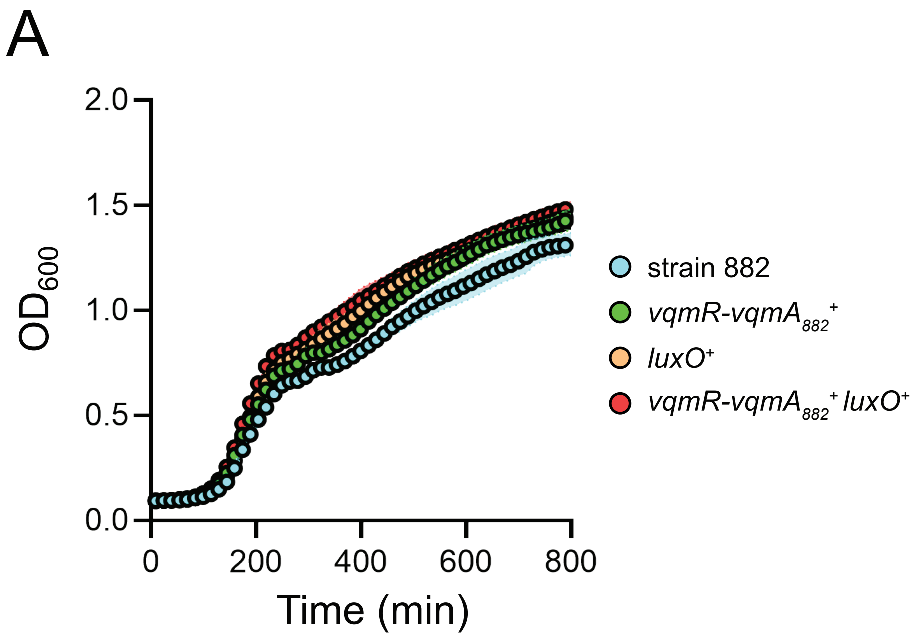

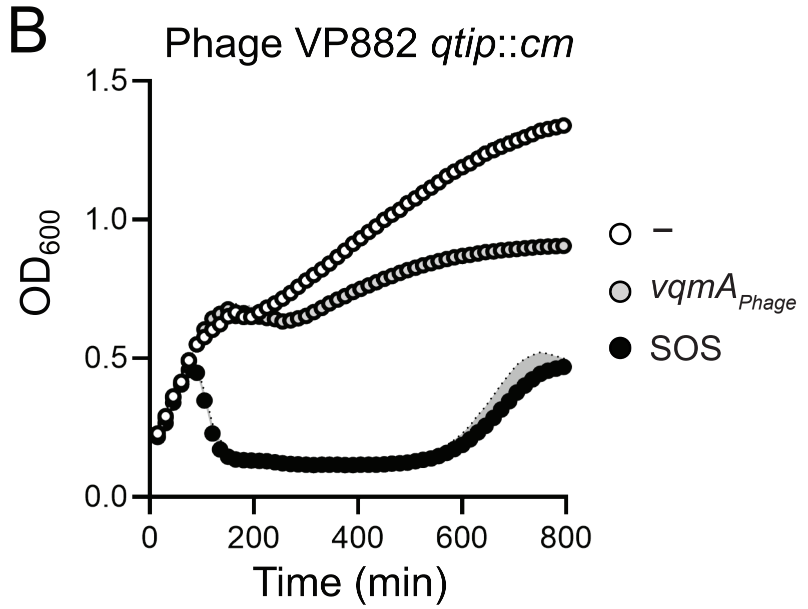

bioRxiv preprint doi: https://doi.org/10.1101/2023.06.05.543668; this version posted June 5, 2023. The copyright holder for this preprint (which was not certified by peer review) is the author/funder, who has granted bioRxiv a license to display the preprint in perpetuity. It is made available under aCC-BY 4.0 International license.

Supplementary Figure 4. There are no growth defects in the QS-active 882 strains carrying the phage and elimination of *qtip* renders phage VP882 SOS-inducible but not $\text{VqmA}_{\text{Phage}}$-inducible.

(A) Growth of the 882 parent (cyan), 882 *vqmR-vqmA*$_{882}^{+}$ (green), 882 *luxO*$^{+}$ (orange), and 882 *vqmR-vqmA*$_{882}^{+}$ *luxO*$^{+}$ (red) strains.
(B) Growth of strain 882 harboring phage VP882 *qtip*::*cm* and arabinose-inducible *vqmA*$_{Phage}$ in medium treated with water (white), arabinose (gray), or ciprofloxacin (black). Arabinose (0.2%) was used to induce *vqmA*$_{Phage}$ expression, and ciprofloxacin (500 ng mL^{-1}) was used to induce host SOS.
Data are represented as means ± std with *n* = 3 biological replicates (A, B).

bioRxiv preprint doi: https://doi.org/10.1101/2023.06.05.543668; this version posted June 5, 2023. The copyright holder for this preprint (which was not certified by peer review) is the author/funder, who has granted bioRxiv a license to display the preprint in perpetuity. It is made available under aCC-BY 4.0 International license.

Table S1 Numerical values, and associated p-values, for heatmaps in Figure 4A.

	vqmR-vqmA$_{882}$$^+$		***luxO$^+$***		***vqmR-vqmA$_{882}$$^+$ luxO$^+$***	
VP882 gene	log_2FC	P-value	log_2FC	P-value	log_2FC	P-value
gp01	0.868	8.52E-02	3.282	3.76E-10	3.368	1.86E-10
gp04	0.467	2.85E-03	2.778	1.35E-20	3.835	5.68E-25
gp05	0.351	1.88E-02	2.648	3.47E-20	3.402	1.37E-23
gp06	0.460	7.41E-04	2.499	1.68E-21	3.147	9.66E-25
gp07	0.551	3.42E-04	2.643	1.59E-20	3.352	8.66E-24
gp10	0.539	8.49E-05	2.842	1.08E-23	3.399	3.33E-26
gp11	0.529	2.87E-04	2.758	5.01E-22	3.452	3.86E-25
gp14	0.490	5.77E-03	2.243	6.61E-15	2.920	5.26E-18
gp16	0.443	2.37E-02	2.061	1.16E-14	2.664	1.59E-18
gp17	0.378	6.54E-03	2.149	3.81E-19	2.794	5.86E-23
gp18	0.440	1.03E-03	2.007	6.81E-19	2.714	2.85E-23
gp19	0.541	5.06E-04	2.230	6.66E-18	2.897	1.82E-21
gp20	0.532	1.72E-04	2.216	1.41E-19	2.812	6.13E-23
gp21	0.499	1.03E-03	2.396	3.12E-19	2.913	6.09E-22
gp22	0.497	1.84E-04	2.384	1.40E-21	2.980	9.09E-25
gp23	0.643	1.13E-04	2.371	1.07E-17	3.099	3.66E-21
gp24	0.395	3.98E-03	2.223	1.81E-19	2.750	1.85E-22
gp25	0.321	7.45E-03	2.382	2.36E-22	2.490	5.45E-23
gp26	0.415	2.23E-02	2.260	5.52E-14	2.447	7.62E-15
gp27	0.516	1.38E-03	2.443	5.13E-18	2.695	2.77E-19
gp28	0.552	1.51E-03	2.537	1.42E-17	3.152	2.19E-20
gp29	0.595	4.68E-05	2.842	9.15E-23	3.306	7.26E-25
gp30	0.489	1.67E-03	2.106	7.77E-17	2.614	1.01E-19
gp31	0.576	4.80E-04	2.078	3.43E-16	2.907	1.03E-20
gp32	0.478	2.90E-04	1.924	1.36E-18	2.676	2.58E-23
gp33	0.277	2.68E-01	1.183	9.93E-06	2.155	5.05E-12
gp35	0.478	3.74E-04	1.832	1.38E-17	2.321	6.13E-21
gp36	0.317	1.87E-01	1.510	2.31E-07	1.890	2.59E-09
gp37	0.413	4.50E-04	2.461	1.01E-23	2.679	5.96E-25
gp38	0.283	1.89E-02	2.371	3.94E-22	2.615	1.54E-23
gp39	0.385	6.27E-03	2.098	3.00E-18	2.977	3.66E-23
gp42	0.457	1.11E-01	1.842	8.21E-09	2.012	8.44E-10
gp45	0.018	8.93E-01	-0.344	1.19E-02	-0.231	8.44E-02
gp46	0.224	3.05E-02	-0.587	7.95E-07	-0.532	4.62E-06
gp47	-0.119	3.19E-01	-0.702	7.74E-07	-0.865	1.20E-08
gp48	0.134	4.25E-01	-0.450	1.27E-02	-0.525	4.51E-03
gp49	0.203	1.75E-01	-0.670	9.08E-05	-0.166	2.79E-01
gp51	0.340	6.25E-01	-1.221	1.90E-01	0.543	4.53E-01
gp52	0.149	2.48E-01	0.599	2.95E-05	0.794	2.19E-07
parA	0.247	4.25E-02	0.804	3.83E-08	0.828	2.03E-08
telN	0.481	2.51E-04	0.019	8.76E-01	0.183	1.34E-01
qtip	0.156	5.66E-01	0.467	9.03E-02	0.705	1.19E-02
vqmA$_{Phage}$	0.066	6.48E-01	1.277	5.71E-11	1.796	3.29E-15
repA	0.272	1.77E-02	1.757	1.51E-18	2.179	1.23E-21
cI	-0.040	7.25E-01	-0.159	1.68E-01	0.180	1.19E-01
putative *cro*	0.210	9.26E-02	1.668	1.74E-16	2.110	8.64E-20
putative *dskA*	0.528	8.35E-04	1.861	4.85E-16	2.093	1.03E-17
q	0.159	2.18E-01	1.485	1.04E-14	1.738	6.95E-17
unknown	0.148	2.89E-01	0.092	5.12E-01	0.424	3.98E-03
gp69 lysin	0.234	6.33E-02	2.465	8.39E-22	3.091	5.03E-25
gp70 lysin	0.336	5.75E-03	2.686	6.14E-24	3.146	3.45E-26
gp71 lysin	0.207	9.20E-02	2.305	4.35E-21	2.723	1.83E-23

bioRxiv preprint doi: https://doi.org/10.1101/2023.06.05.543668; this version posted June 5, 2023. The copyright holder for this preprint (which was not certified by peer review) is the author/funder, who has granted bioRxiv a license to display the preprint in perpetuity. It is made available under aCC-BY 4.0 International license.

Table S2 Strains used in this study.

Strain	Genotype	Reference
V. cholerae str. C6706	*vqmA*$_{Vc}$::*vqmA*$_{Vc}$*-3XFLAG*	[7]
	P*vqmA*$_{RIMD}$*-vqmA*$_{Vc}$*-3XFLAG*	This study
E. coli BW25113	*lacIq*, *rrnBT14*, Δ*lacZWJ16*, *hsdR514*, Δ*araBADAH33*, Δ*rhaBADLD78*, Δ*tdh*	[8]
E. coli S17λ*pir*	Δ*lacU169 (ΦlacZ*Δ*M15), recA1, endA1, hsdR17, thi-1, gyrA96, relA1, λpir*	[34]
E. coli TOP10	F- *mcrA* Δ(*mrr-hsdRMS-mcrBC*) Φ80*lacZ*Δ*M15* Δ*lacX74 recA1 araD139* Δ(*ara leu*)*7697 galU galK rpsL* (Str^R) *endA1 nupG*	Invitrogen
V. parahaemolyticus O3:K6 RIMD2210633	*vqmA*$_{RIMD}$::*vqmA*$_{RIMD}$*-3XFLAG*	This study
	P*vqmA*$_{Vc}$*-vqmA*$_{RIMD}$*-3XFLAG*	This study
V. parahaemolyticus O3:K6 strain 882	*vqmA*$_{882}$::*vqmA*$_{882}$*-3XFLAG* (882 parent)	This study
	P*vqmA*$_{882}$*-vqmA*$_{882}$::*vqmA*$_{882}$*-3XFLAG (vqmA*$_{882}^{+}$)	This study
	vqmR^{+}-P*vqmA*$_{882}$*-vqmA*$_{882}$::*vqmA*$_{882}$*-3XFLAG (vqmR-vqmA*$_{882}^{+}$)	This study
	vqmA$_{882}$::*vqmA*$_{882}$*-3XFLAG; luxO*$_{882}$::*luxO*$_{RIMD}$ (*luxO*$^{+}$)	This study
	vqmR^{+}-P*vqmA*$_{882}$*-vqmA*$_{882}$::*vqmA*$_{882}$*-3XFLAG; luxO*$_{882}$::*luxO*$_{RIMD}$ (*vqmR-vqmA*$_{882}^{+}$ *luxO*$^{+}$)	This study
	vqmA$_{882}$::*vqmA*$_{882}$*-3XFLAG; luxO*$_{882}$::*luxO*^{D61E} (*luxO*^{D61E})	This study
	vqmA$_{882}$::*vqmA*$_{882}$*-3XFLAG; luxO*$_{882}$::*luxO*^{D61A} (*luxO*^{D61A})	This study
	vqmA$_{882}$::*vqmA*$_{882}$*-3XFLAG; luxO*$_{882}$::*luxO*$_{882}^{D61E}$ (*luxO*$_{882}^{D61E}$)	This study
	vqmA$_{882}$::*vqmA*$_{882}$*-3XFLAG; luxO*$_{882}$::*luxO*$_{882}^{D61A}$ (*luxO*$_{882}^{D61A}$)	This study
	vqmA$_{882}$::*vqmA*$_{882}$*-3XFLAG; luxO*$_{882}$::*luxO*$_{882}$ (*luxO-3XFLAG*)	This study
	vqmA$_{882}$::*vqmA*$_{882}$*-3XFLAG; luxO*$_{882}$::*luxO*$_{RIMD}$*-3XFLAG* (*luxO*$^{+}$*-3XFLAG*)	This study

bioRxiv preprint doi: https://doi.org/10.1101/2023.06.05.543668; this version posted June 5, 2023. The copyright holder for this preprint (which was not certified by peer review) is the author/funder, who has granted bioRxiv a license to display the preprint in perpetuity. It is made available under aCC-BY 4.0 International license.

Table S3 Primers and gBlocks used in this study.

Name	Sequence (5 - 3)	Purpose; Template
ODO-786	CTGCTGACTCTGATTGTGCTG	qPCR; phage VP882 *gp69*
ODO-787	TCGTGAGAGGTGATGTACTTCTC	qPCR; phage VP882 *gp69*
ODO-30	CTAAGGGGCAATCTCTACAAGACCCA	qPCR; strain 882 *hfq*
ODO-370	ACCATTTGGTTTACAGTATTTTTCAGCAG	qPCR; strain 882 *hfq*
ODO-943	TTCAAACAGATCCTGAAGCGCCTC	First-round amplification VP882 *qtip* 3Kb upstream flanking sequence; from strain 882 gDNA
ODO-944	TACTGCGATGAGTGGCAGGGCGGGGCGTAAGAT TAGTCATCGAGTGCCTTTTGGCTG	First-round amplification VP882 *qtip* 3Kb upstream flanking sequence; from strain 882 gDNA
ODO-947	GGCGTGGTCCGCCCGAGGGCAGAGCCATGACA CACGAATACACTCCTTGTAAGTGATTGT	First-round amplification VP882 *qtip* 3Kb downstream flanking sequence; from strain 882 gDNA
ODO-948	TCAACTACATCCGCCTCGAGGG	First-round amplification VP882 *qtip* 3Kb downstream flanking sequence; from strain 882 gDNA
ODO-945	TTACGCCCCGCCCTGCCACTCA	First-round amplification of *cat-traJ*; Ec-OD603
ODO-946	TCATGGCTCTGCCCTCGGGCGGAC	First-round amplification of *cat-traJ*; Ec-OD603
ODO-949	AATTGGCAGCTCGTACCCTTC	Second round amplification to construct *qtip*::*cm*; ODO-943x944, ODO-947x948, ODO-9454x946
ODO-950	GACACTCTTTGCATCGATGAGGCC	Second round amplification to construct *qtip*::*cm*; ODO-943x944, ODO-947x948, ODO-9454x946
ODO-951	TCATGGCTCTCAAGACTTTTTGATCA	Intromolecular reclosure to delete Δ91-102 *luxO;* FJS-S113 and FJS-S114
ODO-952	GCCGTCATGAAAATGATCGGCA	Intromolecular reclosure to delete Δ91-102 *luxO;* FJS-S113 and FJS-S114
pRE112-1	ATGCAGTTCACTTACACCGCTTC	Amplification of pRE112 backbone; pRE112 plasmid
pRE112-2	GGGATCGGGCCCTATCACTT	Amplification of pRE112 backbone; pRE112 plasmid
ODO-651	GAAGCGGTGTAAGTGAACTGCATATCAGTTCGAT GATGTTCGAATACCGC	Amplification to construct $luxO_{RIMD}$ in pRE112; from *V. parahaemolyticus* RIMD2210633 gDNA
ODO-656	AAGTGATAGGGCCCGATCCCTGTTACGACTCTG ACCAAACGAATGG	Amplification to construct $luxO_{RIMD}$ in pRE112; from *V. parahaemolyticus* RIMD2210633 gDNA
ODO-619	GTGAGTCCTCTTGTGAATATTCCAGTCAA	Amplification of pRE112 backbone containing $vqmA_{RIMD}$*-3XFLAG*; pRE112-$vqmA_{RIMD}$*-3XFLAG*
ODO-620	GGATCAAAAAAAAAGCCAGCCTGAAGA	Amplification of pRE112 backbone containing $vqmA_{RIMD}$*-3XFLAG*; pRE112-$vqmA_{RIMD}$*-3XFLAG*
ODO-621	TCTTCAGGCTGGCTTTTTTTTTGATCCAAACAGTC CCCGAATAGGCAA	Amplification of $vqmA_{Vc}$ promoter to construct $PvqmA_{Vc}$-$vqmA_{RIMD}$*-3XFLAG*; from *V. cholerae* gDNA

bioRxiv preprint doi: https://doi.org/10.1101/2023.06.05.543668; this version posted June 5, 2023. The copyright holder for this preprint (which was not certified by peer review) is the author/funder, who has granted bioRxiv a license to display the preprint in perpetuity. It is made available under aCC-BY 4.0 International license.

ODO-622	TTGACTGGAATATTCACAAGAGGACTCACGCCAT ATCCTCCACTGGAAATGCG	Amplification of $vqmA_{Vc}$ promoter to construct $PvqmA_{Vc}$-$vqmA_{RIMD}$-*3XFLAG*; from *V. cholerae* gDNA
ODO-626	GCTTATGCCATTGTTTTCATTTTTGAGATAGAAAA AAAGCCAGCCTGAAGACGG	Amplification of pKAS backbone containing $vqmA_{Vc}$-*3XFLAG*; pKAS-$vqmA_{Vc}$-*3XFLAG* (pKP485)
ODO-629	TGGGGCCAAATAGCAGTAGTGAGTCCTCTTGTG CCTAACCATCTGACATTAG	Amplification of pKAS backbone containing $vqmA_{Vc}$-*3XFLAG*; pKAS-$vqmA_{Vc}$-*3XFLAG* (pKP485)
ODO-627	CCGTCTTCAGGCTGGCTTTTTTTCTATCTCAAAAA TGAAAACAATGGCATAAGC	Amplification of $vqmA_{RIMD}$ promoter to construct P$vqmA_{RIMD}$-$vqmA_{Vc}$-*3XFLAG*; from *V. parahaemolyticus* RIMD2210633 gDNA
ODO-912	TACTGCTATTTGGCCCCAAGCC	Amplification of $vqmA_{RIMD}$ promoter to construct P$vqmA_{RIMD}$-$vqmA_{Vc}$-*3XFLAG*; from *V. parahaemolyticus* RIMD2210633 gDNA
ODO-427	TTAATTAACTCGAGCGGTACCCGCCG	Amplification of the pEVS backbone containing luciferase genes; P$vqmR_{Vc}$-lux plasmid (EcOD119)
ODO-426	ATGACTAAAAAAATTTCATTCATTATTAACGGCCA GG	Amplification of the pEVS backbone containing luciferase genes; P$vqmR_{Vc}$-lux plasmid (EcOD119)
ODO-860	CGGCGGGTACCGCTCGAGTTAATTAAATGTGAG ATTAACTTACGGTCAATGTACAA	Amplification of $vqmR_{RIMD}$ promoter to insert into pEVS-*lux;* from *V. parahaemolyticus* RIMD2210633 gDNA
ODO-558	CCTGGCCGTTAATAATGAATGAAATTTTTTTAGTC ATCAGGGTCGTATGCATCCTAGAGC	Amplification of $vqmR_{RIMD}$ promoter to insert into pEVS-*lux;* from *V. parahaemolyticus* RIMD2210633 gDNA
ODO-930	CGGCGGGTACCGCTCGAGTTAATTAAAGCATCAT CCCCTTCGCTTATTGA	Amplification of *qtip* promoter to insert into pEVS-*lux*; from strain 882 gDNA
ODO-931	CCTGGCCGTTAATAATGAATGAAATTTTT	Amplification of *qtip* promoter to insert into pEVS-*lux*; from strain 882 gDNA
ODO-551	GGGTTGAGAAGCGGTGTAAGTGAACTGCATAAT CACAATCTGCGTGTAAGCATGATCA	Amplification of $vqmA_{RIMD}$ upstream sequence to construct $vqmA_{RIMD}$-*3XFLAG*; from *V. parahaemolyticus* RIMD2210633 gDNA
ODO-552	ACGCCTGAATAAGTGATAGGGCCCGATCCCTCA CGTTACTTCTCGTCGCTTCATTCT	Amplification of $vqmA_{RIMD}$ *and* $vqmA_{882}$ downstream sequence to construct $vqmA_{RIMD}$ - and $vqmA_{882}$ -*3XFLAG*; from strain 882 gDNA
ODO-553	GGGTTGAGAAGCGGTGTAAGTGAACTGCATCAC AACAAAATGTTAGGAATATCAATAAGA	Amplification of $vqmA_{882}$ upstream sequence to construct $vqmA_{882}$-*3XFLAG* in pRE112; from strain 882 gDNA
ODO-586	TACTGCTATTTGGCCCCAAGCCATC	Amplification of $vqmA_{RIMD}$ *and* $vqmA_{882}$ upstream sequence to construct $vqmA_{RIMD}$ - and $vqmA_{882}$ -*3XFLAG*; from strain 882 and *V. parahaemolyticus* RIMD2210633 gDNA
ODO-529	CGCCACGCCGCTTTATCTTTATAAACA	Amplification of $vqmA_{RIMD}$ *and* $vqmA_{882}$ downstream sequence to construct $vqmA_{RIMD}$ - and $vqmA_{882}$ -*3XFLAG*; from *V. parahaemolyticus* RIMD2210633 gDNA

bioRxiv preprint doi: https://doi.org/10.1101/2023.06.05.543668; this version posted June 5, 2023. The copyright holder for this preprint (which was not certified by peer review) is the author/funder, who has granted bioRxiv a license to display the preprint in perpetuity. It is made available under aCC-BY 4.0 International license.

ODO-1	GCCATATCCTCCACTGGAAATGC	Amplification of pBR322-pBAD backbone; pBAD-*vqmA*$_{Vc}$-*3XFLAG* (BB-Ec0042)
ODO-2	TAAGCAACAACGTCAAGCTGATTG	Amplification of pBR322-pBAD backbone; pBAD-*vqmA*$_{Vc}$-*3XFLAG* (BB-Ec0042)
ODO-635	GCATTTCCAGTGGAGGATATGGCGTGAGTCCTC TTGTGAATATTCCAG	Amplification of *vqmA*$_{RIMD}$ to insert into pBR322-pBAD; *vqmA*$_{RIMD}$-*3XFLAG* gBlock
ODO-636	CAATCAGCTTGACGTTGTTGCTTATTATTTATCGT CATCTTTGTAGTCGATATCATG	Amplification of *vqmA*$_{RIMD}$ to insert into pBR322-pBAD; *vqmA*$_{RIMD}$-*3XFLAG* gBlock
pEVS-P*vqmA*$_{RIMD}$ 2	CGGCGGGTACCGCTCGAGTTAATTAATCGAGCT CTAGGATGCATACGACCCTGAGCTCTCGGTATCT GATTGATACCTCTAACTTACTTAGCAGACCCTCG CTATGTAATTTAGAACCTCTTCTACACGTCGACAA GATTATATCTTGTGTTGCCAGCCCGTCTTCAGGC TGGCTTTTTTTTTGTATCTCAAAAATGAAAACAAT GGCATAAGCTGATATTATATCGGCCATCTCACCG TATCCTTACGTCAATCACCACGCTGAGTACTCTT GATCCATTTTTCTCCGCTCCCAGTGCCATCTGGT GCTATTTACCGAAAGCATGATCAAGGAATAACAC TGCGTAGATGACGAAAGAATCCATGAAGTGAGC ATACTGACCTGCATAAAGCAGGCAATGTATCCAA GTCATGTCTGAATGCTGGTGTAAACACAGCTGAT GGAGAGATGGCTTGGGGCCAAATAGCAGTAATG ACTAAAAAAATTTCATTCATTATTAACGGCCAGG	P*vqmA*$_{RIMD}$ for insertion into pEVS-*lux*
ODgBlock12	GATGGCTTGGGGCCAAATAGCAGTAGTGAGTCC TCTTGTGAATATTCCAGTCAATACCATAGAACAGT CGCTCCTCCGACAGCTACCGGGTTGTTGGGGGT GCAAAGATAAAGATTCGGTATTCCGTTATGTGAA TCAGGAATACGCCGAATTATTGGGCCATGCCTCA CCAGAAGAGTGCATTGGTAAAACTGACTTCGAGA TGTCGAGCCCAACCACTGAATGCGCACAAGAATT TCAACGCCAAGATAAACATGTGATCGAAACGGG CGAATCGTTGAAGATCCTTGATATTCATCCTTAC CCTGATGGGCGTTGGCGCGCACATATTTTCACG AAAACCCCTTGGCGAGATGAACAAGGCAACACC CTCGGCACCATTTTTTATGGCCGAGAGTTAACCG ACACCGCCGTGATTGAAGTGGGCTACTGGGTGT GTCGAGCCATCGGTACAGACATGAACCATCAATC CATTTTCCGCTTTTCGAATCTGAATCCGAAACCA GAAAAGCTCACATGCCGCGAGCAAGAAACGCTA TTTTTACTGCTTTACGGCAAAAAACCTCAGTTTAT TTCTCAAGTGATGGGCATCTCCACAAAAACGGTA GAAGGGCACGTCGCACGTCTAAGAAACAAGTTT GAAGCGAATAGTAAGAACGAGCTGATCGATAAA GCAATGGAGGCAGGCTATGGATCTGTTGTACCC AAAACGCTGCTTAAACATCAGCTTTCTGTTGTTTT GAATGGAGAGCGCGACTACAAAGACCATGACGG TGATTATAAAGATCATGATATCGACTACAAAGATG ACGATAAATAACGCCACGCCGCTTTATCTTTATA AACA	*vqmA*$_{RIMD}$-*3XFLAG* insert to make *V. parahaemolyticus vqmA-3XFLAG* pRE112 plasmids and pBAD-*vqmA*$_{RIMD}$-*3XFLAG*

bioRxiv preprint doi: https://doi.org/10.1101/2023.06.05.543668; this version posted June 5, 2023. The copyright holder for this preprint (which was not certified by peer review) is the author/funder, who has granted bioRxiv a license to display the preprint in perpetuity. It is made available under aCC-BY 4.0 International license.

ODgBlock18	TTTTTTGTTGATTCAATATCGTCATGCTTATTTATC<br>GTCATCTTTGTAGTCGATATCATGATCTTTATAAT<br>CACCGTCATGGTCTTTGTAGTCCACCTTCTGCCG<br>CTCTTCTTTACCATTCCATGCTTGTAGTTTGCGGT<br>AAATCGTTGACGGACTGACATCAAGATACCCAGC<br>GGCTCTTGGAATATTGCCGTCACACGCCTCAATC<br>GCTTGCTCAATCGCCATTTTCTCTGTCATCCATA<br>GCGGCATGATCTCTGAAACAGTCATCGCTTTTGG<br>CTCAATCAGTTTAGATACAGATGGCCTATCTAAC<br>GGTTGATTCAGCGGCGGTGGCAACATATCTAAC<br>GTAATTTCTTTGCCGTTGTTGAGCACCACGATAT<br>TGCGTAGTACGTTTTGTAGTTGTCGGACGTTACC<br>CGGCCACTCATAACTGTTAAAGCGGTCAATCACT<br>TCTTGAGAAAAGCGGACAAAGTTTTTGCCCTCTT<br>CATGAGACATGTAGCCTAACAGTGAGTACGCAAT<br>TTCAATCACATCTTCACCACGTTCGCGAAGAGGC<br>GGAAGATGCAAAGGAATCACGTATAAACGGTAGT<br>ATAAGTCTTCGCGGAAACGCCCTTCTTGAACTTC<br>TTTCCAAGGGTCTCGGTTGGTCGCACAAACGAA<br>GCGCACGTCCACACTCTTCATTTTTGAAGAGCCC<br>ACTTTCTGGAACGTACCGGTTTGGATGAATCGCA<br>ATAATTTGGTTTGTAAGTCCAAATCCATTTCACAC<br>AATTCATCGAGGAACAACGTGCCACCATCGGCTA<br>ACTCAGCGGCACCTTGACGGTCATTTGCCGCAC<br>CAGTAAATGCCCCTTTTACGTGACCAAATAGTTC<br>ACTTTCGATTAAGTCTTTAGGTATGGCCGCACAG<br>TTGATGGCAATAAACGGCTTATCACCACGTTTGC<br>TTGCAGCGTGGATGGCTTCTGCGCATACCTCTTT<br>ACCCGTACCACTTTCACCAGTAATAAAGATACTC<br>GCTTTACTGCTAGCGGCAGAGTCAATGGTGCGA<br>TAGACCTGCTGCATGGTTTGGCTGCTGCCGATAA<br>AACCTTGGTAATTCTGATTACCCGGATTATCTGC<br>TTCATTTTTTAGCTTAGTTGCTTTGCGAATTGCGT<br>TGTTGACCGTAACACGCAGACGGTCTGCTTCACA<br>CGGTTTGATCAAAAAGTCTTGAGAGCCATGACGC<br>ATCGCTTCTACCGCTGTATCGATAGAGCCATGAG<br>CCGTCATGAAAATGATCGGCACATCTGGATGACT<br>CTTTTTAACGGCGTGCAAAACATCCATCCCCGTC<br>ATATCTGGCAGACGAAGATCGAGAAGAATAAGAT<br>CTGGAATTCGATGATTGAGACTTTCAATGGCATC<br>TCTACCTGTACCGACAATATTAATGTCGATCCCT<br>AACGGCGTGAGGTACGAACGGTATAACGCCGCA<br>ACCGATGCTGTATCCTCAACCAT	*luxO*$_{RIMD}$*-3XFLAG* for insertion into pRE112

bioRxiv preprint doi: https://doi.org/10.1101/2023.06.05.543668; this version posted June 5, 2023. The copyright holder for this preprint (which was not certified by peer review) is the author/funder, who has granted bioRxiv a license to display the preprint in perpetuity. It is made available under aCC-BY 4.0 International license.

ODgBlock19	TTTTTTGTTGATTCAATATCGTCATGCTTATTTATC<br>GTCATCTTTGTAGTCGATATCATGATCTTTATAAT<br>CACCGTCATGGTCTTTGTAGTCCACCTTCTGCCG<br>CTCTTCTTTACCATTCCATGCTTGTAGTTTGCGGT<br>AAATCGTTGACGGACTGACATCAAGATACCCAGC<br>GGCTCTTGGAATATTGCCGTCACACGCCTCAATC<br>GCTTGCTCAATCGCCATTTTCTCTGTCATCCATA<br>GCGGCATGATCTCTGAAACAGTCATCGCTTTTGG<br>CTCAATCAGTTTAGATACAGATGGCCTATCTAAC<br>GGTTGATTCAGCGGCGGTGGCAACATATCTAAC<br>GTAATTTCTTTGCCGTTGTTGAGCACCACGATAT<br>TGCGTAGTACGTTTTGTAGTTGTCGGACGTTACC<br>CGGCCACTCATAACTGTTAAAGCGGTCAATCACT<br>TCTTGAGAAAAGCGGACAAAGTTTTTGCCCTCTT<br>CATGAGACATGTAGCCTAACAGTGAGTACGCAAT<br>TTCAATCACATCTTCACCACGTTCGCGAAGAGGC<br>GGAAGATGCAAAGGAATCACGTATAAACGGTAGT<br>ATAAGTCTTCGCGGAAACGCCCTTCTTGAACTTC<br>TTTCCAAGGGTCTCGGTTGGTCGCACAAACGAA<br>GCGCACGTCCACACTCTTCATTTTTGAAGAGCCC<br>ACTTTCTGGAACGTACCGGTTTGGATGAATCGCA<br>ATAATTTGGTTTGTAAGTCCAAATCCATTTCACAC<br>AATTCATCGAGGAACAACGTGCCACCATCGGCTA<br>ACTCAGCGGCACCTTGACGGTCATTTGCCGCAC<br>CAGTAAATGCCCCTTTTACGTGACCAAATAGTTC<br>ACTTTCGATTAAGTCTTTAGGTATGGCCGCACAG<br>TTGATGGCAATAAACGGCTTATCACCACGTTTGC<br>TTGCAGCGTGGATGGCTTCTGCGCATACCTCTTT<br>ACCCGTACCACTTTCACCAGTAATAAAGATACTC<br>GCTTTACTGCTAGCGGCAGAGTCAATGGTGCGA<br>TAGACCTGCTGCATGGTTTGGCTGCTGCCGATAA<br>AACCTTGGTAATTCTGATTACCCGGATTATCTGC<br>TTCATTTTTTAGCTTAGTTGCTTTGCGAATTGCGT<br>TGTTGACCGTAACACGCAGACGGTCTGCTTCACA<br>CGGTTTGATCAAAAAGTCTTGAGAGCCATGAGCC<br>GTCATGAAAATGATCGGCACATCTGGATGACTCT<br>TTTTAACGGCGTGCAAAACATCCATCCCCGTCAT<br>ATCTGGCAGACGAAGATCGAGAAGAATAAGATCT<br>GGAATTCGATGATTGAGACTTTCAATGGCATCTC<br>TACCTGTACCGACAATATTAATGTCGATCCCTAA<br>CGGCGTGAGGTACGAACGGTATAACGCCGCAAC<br>CGATGCTGTATCCTCAACCAT	*luxO*$_{882}$-*3XFLAG* for insertion into pRE112

bioRxiv preprint doi: https://doi.org/10.1101/2023.06.05.543668; this version posted June 5, 2023. The copyright holder for this preprint (which was not certified by peer review) is the author/funder, who has granted bioRxiv a license to display the preprint in perpetuity. It is made available under aCC-BY 4.0 International license.

Table S4 Plasmids used in this study.

Plasmid name (informal)	Strain ID (formal)	Marker, Origin	Source
VP882 Ctr::*cm*	Ec-OD603	Cm, VP882	This study, courtesy of G. Beggs
VP882 *qtip*::*cm*	Ec-OD587	Cm, VP882	This study
P*gp69-lux*	JSS-1220	Kan, p15A	[8]
P*qrr3-mRuby3*	BB-Ec0914	Cm, p15A	[32]
P*luxC-CDABE*	BB-Ec0224	Tet, pLAFR	[14]
pBAD-$vqmA_{Phage}$	JSS-852	Kan, p15A	[8]
HIS-HALO-cI_{VP882}-P*q-lux*	JSS-3410	Amp, Kan, pBR322	This study
P$vqmR_{Vc}$-*lux*	Ec-OD119	Kan, p15A	[8]
P$vqmR_{RIMD}$-*lux*	Ec-OD545	Kan, p15A	This study
P$vqmA_{RIMD}$-*lux*	Ec-OD389	Kan, p15A	This study
P*qtip-lux*	Ec-OD567	Kan, p15A	This study
pBAD-$vqmA_{RIMD}$-*3XFLAG*	Ec-OD545	Amp, pBR322	This study
pBAD-$vqmA_{Vc}$-*3XFLAG*	BB-Ec0042	Amp, pBR322	[13]
pRE112-$vqmA_{882}$-*3XFLAG*	Ec-OD498	Cm, oriR6kγ	This study
pRE112-$vqmA_{RIMD}$-*3XFLAG*	Ec-OD499	Cm, oriR6kγ	This study
pRE112-P$vqmA_{882}$-$vqmA_{882}$-*3XFLAG*	Ec-OD500	Cm, oriR6kγ	This study
pRE112-$vqmR^{+}$- P$vqmA_{882}$-$vqmA_{882}$-*3XFLAG*	Ec-OD501	Cm, oriR6kγ	This study
pRE112- P$vqmA_{Vc}$-$vqmA_{RIMD}$-*3XFLAG*	Ec-OD521	Cm, oriR6kγ	This study
pRE112- $luxO_{882}$::$luxO_{RIMD}$	Ec-OD525	Cm, oriR6kγ	This study
pRE112-$luxO^{D61E}$	FJS-S113	Cm, oriR6kγ	This study, courtesy of F. Santoriello
pRE112-$luxO^{D61A}$	FJS-S114	Cm, oriR6kγ	This study, courtesy of F. Santoriello
pRE112-$luxO_{882}{}^{D61E}$	Ec-OD599	Cm, oriR6kγ	This study
pRE112-$luxO_{882}{}^{D61A}$	EcOD-600	Cm, oriR6kγ	This study
pRE112- $luxO_{882}$::$luxO_{RIMD}$-*3XFLAG*	EcOD-601	Cm, oriR6kγ	This study
pRE112- $luxO_{882}$::$luxO_{882}$-*3XFLAG*	EcOD-602	Cm, oriR6kγ	This study
pKAS- P$vqmA_{RIMD}$-$vqmA_{Vc}$-*3XFLAG*	EcOD-550	Amp, oriR6kγ	This study
